# Assessment of Hidden Nutritional Burden: High Prevalence of Disease-Related Malnutrition in Older Adults Without Cognitive Impairment Living in Nursing Homes in Madrid—A Multicentre Study

**DOI:** 10.3390/nu17213325

**Published:** 2025-10-22

**Authors:** Mar Ruperto, Dilek Ongan, Esmeralda Josa, Amalia Tsagari

**Affiliations:** 1 Department of Pharmaceutical & Health Sciences, School of Pharmacy, Universidad San Pablo-CEU, CEU Universities, Urbanización Monteprincipe, 28660 Madrid, Spain; 2 European Specialist Dietetic Network for Older Adults Group, European Federation of the Associations of Dietitians, 1411 Naarden, The Netherlands; mmar.rupertolopez@ceu.es (M.R.); dilek.ongan@ikc.edu.tr (D.O.); tsagaridiet@yahoo.gr (A.T.); 3 Department of Nutrition and Dietetics, Faculty of Health Sciences, İzmir Katip Çelebi University, İzmir 35620, Türkiye; 4 Department of Nutrition and Bromatology, Universidad Complutense de Madrid, Av. Complutense, s/n, Moncloa—Aravaca, 28040 Madrid, Spain; e.josa.martin@gmail.com; 5 KAT General Hospital of Attica, 145 61 Athens, Attica, Greece

**Keywords:** disease-related malnutrition, EuroQol visual analogue scale, GLIM criteria, older people, Mini-Nutritional Assessment, nursing homes

## Abstract

**Background/Objectives:** Nutritional disorders are common conditions in older people. This study aimed to determine nutritional disorders in a Mediterranean cohort of nursing home residents without cognitive or functional impairment. **Methods:** A multicentre cross-sectional observational study was conducted in 10 Spanish geriatric centres. Socio-health, clinical, and laboratory data were recorded from the participants’ medical records. The Mini-Nutritional Assessment (MNA) and Global Leadership Initiative in Nutrition (GLIM) diagnostic criteria [weight loss and serum C-reactive protein (CRP)] were used. Frailty risk was assessed using the FRAIL questionnaire. Anthropometric parameters [body mass index, weight loss, triceps skinfold thickness (TSF), muscle mass circumference (MAMC), and calf-circumference] were evaluated. Body composition [hydration pattern, fat-free mass, muscle mass (MM), fat mass, and phase angle (PhA)] was measured by bioelectrical impedance analysis. Laboratory parameters, such as haemoglobin, total lymphocyte count, serum albumin, transferrin, and CRP, were recorded. Participants were classified into two groups: the disease-related malnutrition (DRM) group and the no-DRM group. Using multivariate regression analysis, predictive factors for nutritional status were tested. **Results:** Among 340 participants, 63.2% were over 85 years old, 28.2% were men, and the median length of stay was 24 months (range: 6–119). Nutritional risk or malnutrition, as assessed by the MNA, was present in 60.8% of the residents. DRM was diagnosed in 39.4%, and frailty risk was diagnosed in 57.6%. Older adults with DRM had significantly lower MAMC, calfcircumference, MM, and serum albumin, as well as higher CRP concentrations compared with their No-DRM counterparts (*all, at least*, *p* < 0.05). The frailty risk (OR = 3.317), MM (OR = 0.732), PhA (OR = 0.033), serum albumin (OR = 0.070), and EuroQol visual analogue scale (OR = 0.961) were risk predictors of DRM in nursing home residents. **Conclusions**: This study supports the importance of conducting comprehensive nutritional assessments to ensure the earliest recognition of nutrition disorders in nursing homes. Older adults with DRM had greater unintentional weight loss, inflammation, and a high risk of frailty, as well as reduced MM, compared to those without DRM. Subclinical low-grade systemic inflammation is a risk factor for DRE and functional decline in older adults living in nursing homes. The generalisation of the study results is limited to institutionalised older adults without cognitive impairment who are clinically stable and functionally independent.

## 1. Introduction

The global ageing of the population is one of the main factors contributing to chronic non-communicable diseases, being closely related to the high prevalence of nutritional disorders. By 2050, the proportion of older people (≥65 years) is expected to be 17.0% [1]. This demographic shift underlines the importance of promoting healthy ageing and preventing or treating major comorbidities such as diabetes mellitus (DM), hypertension, obesity, and cardiovascular disease (CVD).

Nutritional disorders are common conditions in older adults, since malnutrition remains frequently underdiagnosed in long-term care facilities [2,3,4,5]. The risk of malnutrition varies considerably between countries and care settings, ranging from 2.8% in the community, 12.9% in hospitals, and 47.6% in nursing homes. Prevalence rates differed by country, from 15.2% in Spain to 37.7% in Switzerland, and by screening tool, from 14.9% using MNA-SF to 40.6% using NRS-2002 [3]. In addition, approximately 399,000 older Spanish adults reside in nursing homes, representing a highly vulnerable subgroup with an elevated risk of nutritional disorders [6,7,8,9], a trend consistent with reports across Europe [3,5,10,11,12]. Factors such as institutionalisation, reduced food intake, multimorbidity, inflammaging, frailty, and cognitive decline increase nutritional risk and mortality rate in older adults [13,14,15,16]. It should be noted that inflammaging is a low-grade systemic inflammatory process caused by various factors such as oxidative stress, cellular senescence, and immune dysregulation, which contributes to the onset of various age-related diseases, such as CVD, neurodegenerative, and metabolic disorders [17].

The Mini Nutritional Assessment (MNA) is a non-invasive, easy-to-use tool recommended for use in nursing homes and other healthcare facilities [18,19]. A meta-analysis reported that approximately 30–60% of older adults are at nutritional risk, and 15–30% are malnourished, according to MNA-based evaluations [5]. Additionally, the Global Leadership Initiative on Malnutrition (GLIM) [20] developed the universal consensus for the diagnosis of disease-related malnutrition (DRM) based on nutritional risk screening tool approaches (i.e., MNA) and the conjoint use of phenotypic [weight loss, body mass index (BMI), muscle mass assessment] and etiological criteria (food intake, malabsorption, inflammation). Some studies applying the GLIM criteria in older adults [4,13,21,22,23] reported high prevalence rates of DRM, particularly among hospitalized cohorts and older individuals living in nursing homes. In a Spanish multicentre cross-sectional cohort of older adults [24], nutritional risk and DRM were strongly associated with higher hospitalisation rates and increased healthcare costs. Similarly, studies conducted in geriatric rehabilitation and long-term care facilities [5,10,25] using GLIM criteria reported DRM up to 50%, depending on comorbidity burden and functional status. To date, few studies have applied the GLIM criteria only to older adults without cognitive impairment in nursing homes. This fact pushes us to assess the nutritional risk factors and DRM in an older Mediterranean cohort of long-term care facilities. Therefore, this study aimed to determine nutritional risk and DRM using the GLIM criteria in a Mediterranean cohort of nursing home residents.

## 2. Materials and Methods

### 2.1. Study Design and Population

This cross-sectional observational multicentre study was conducted in a convenience sample of 340 older adults from 10 geriatric care centres located in Madrid (Spain) between 2023 and 2024.

Participants eligible for the study were over 65 years old with a length of stay (LOS) of at least 3 months in one of the participating nursing homes.

Participants were excluded if they had mild to severe cognitive impairment or dementia, which could interfere with their ability to understand or reliably complete the assessment. Older adults experiencing acute illness, clinical instability, or chronic terminal illnesses at the time of data collection were also excluded to ensure consistency during evaluation. Additionally, any refusal to participate or withdrawal of informed consent at any point before or during the nutritional assessment resulted in exclusion from the study.

All participants signed a written informed consent form before commencing the study, which provided full information about the study’s purpose, procedures, and confidentiality.

The study was conducted in accordance with the Declaration of Helsinki and Good Clinical Practice Guidelines, and it was approved by the Institutional Research Ethics Committee of the San Pablo-CEU University (approval code number: 850/24/113).

### 2.2. Data Collection

Sociodemographic, clinical, and nutritional data were collected through a combination of face-to-face structured interviews, review of medical records, and geriatric comprehensive assessment conducted by the healthcare professional team. The LOS in months was calculated from the date of admission to the facility up to the date of nutritional assessment. Major comorbidities such as DM, arterial hypertension, and dyslipidemia were identified through medical chart reviews or the current use of related medications.

The type of diet for each participant was obtained from their medical records and the institutional menu planning at the nursing home. Participants consuming the regular, unmodified nursing home diet were classified as receiving a standard Mediterranean diet. Oral nutritional supplements were documented based on medical records.

Self-perceived health status was assessed using the EuroQol Visual Analogue Scale (EQ-VAS), a component of the EQ-5D instrument [26,27]. The EQ-VAS ranged from 0 (worst health state) to 100 (best imaginable health state), on which participants reflected their perception of their health on the day of assessment. All collected data were entered into a secure electronic database.

### 2.3. Anthropometric Assessment

Anthropometric parameters such as usual body weight (UBW), BMI, and triceps skinfold thickness (TSF) were measured with standardized methods. The percentage of weight loss was calculated as follows: % weight loss = [(UBW − actual BW) ÷ UBW] × 100, where the actual BW was the resident’s body weight. Mid-arm circumference (MAC) was measured at the midpoint of the non-dominant arm with an inextensible tape measure. TSF was measured by Lange Skin Calipers (Cambridge Instruments, Cambridge, MD, USA). Mid-arm muscle circumference (MAMC) was calculated as follows: MAMC (cm) = MAC (cm) − 0.314 * TSF (mm). MAMC (%) was compared with anthropometric reference values at the 50th percentile for gender, age, and height in the older Spanish population [28]. Calf-circumference (CC) measurements were taken with a non-elastic, flexible measuring tape with the participant in a seated position, with the knee flexed at approximately 90° and the foot flat on the floor to ensure muscle relaxation. Trained clinical dietitians performed all nutritional assessments to ensure inter-observer reliability.

### 2.4. Analysis of Body Composition

Body composition analysis was conducted using a tetrapolar bioelectrical impedance analyser (BIA 101^®^; Akern-RJL Systems, Florence, Italy). The bioelectrical impedance analysis (BIA) test was performed with participants in the supine position, and one disposable electrode was placed on the hand and the other on the back of the foot. Resistance (R), reflecting opposition to current flow through body fluids, and reactance (Xc), reflecting cell membrane capacitance, were measured by BIA to assess body composition. Hydration status was evaluated by measuring total body water (TBW), extracellular water (ECW), intracellular water (ICW), and the exchangeable Na/K ratio. Body composition parameters, including body cell mass (BCM), fat-free mass (FFM), muscle mass (MM), and fat mass (FM), were collected. The BIA-derived phase angle (PhA) was used as an indicator of cellular integrity and functionality, as well as nutritional status.

### 2.5. Laboratory Parameters

Laboratory data, including haemoglobin, total lymphocyte count, serum albumin, transferrin, and serum C-reactive protein (CRP), were obtained retrospectively from participants’ medical electronic records. These parameters were analysed as part of routine clinical evaluations conducted by the nursing home’s medical team. All laboratory tests were performed in certified laboratories using standardized methods.

### 2.6. Nutritional Assessment

The nutritional assessment involved a two-step process: initial screening for malnutrition risk (full-form MNA) [18,19], and the application of GLIM criteria to diagnose DRM [20,29].

The MNA questionnaire [19,30] consists of 18 items based on a multidimensional evaluation that includes anthropometric data, dietary intake, general health, and self-perception of health and nutrition. The total MNA score ranges from 0 to 30 points, classified into three nutritional status groups: well-nourished (24–30 points), at risk of malnutrition (17–23.5 points), and malnourished (<17 points).

Participants identified as being at nutritional risk or malnourished according to the MNA were further evaluated using the GLIM criteria [20]. The diagnosis of DRM was established based on the phenotypic criterion of weight loss ≥ 5% within the last 6 months, in combination with an etiological criterion of CRP ≥ 1 mg/dL. Frailty risk was assessed using the FRAIL [31] screening tool, composed of five self-reported components: Fatigue, Resistance, Ambulation, Illnesses, and weight Loss. The cutoff point for frailty risk was set at ≥3 points.

### 2.7. Statistical Analysis

Data were analysed using descriptive statistics, with quantitative variables expressed as mean and standard deviation, and qualitative variables as frequencies. Chi-square and Fisher’s exact tests were applied to qualitative variables, and Student’s *t*-test to quantitative variables. CRP levels were compared between groups using the Mann–Whitney U test, and results are reported as medians with interquartile ranges (IQRs). Pearson’s Chi-square parametric correlations were examined to assess the strength of the association between the quantitative variables.

Binary logistic regression was performed to assess the relationship between independent variables and the likelihood of being categorized as malnourished (DRM) or no-malnourished (No-DRM), as the dependent variable. Independent variables with a *p*-value ≤ 0.10 were included using forward stepwise regression, adding each variable sequentially. To identify potential confounders in the model, Pearson’s Chi-square correlations and collinearity were examined. A variable was considered a confounder if its inclusion changed the odds ratio (OR) of other variables in the regression model by more than 10%. Statistical analyses were performed using the Statistical Package for the Social Sciences (SPSS) version 28.0 software (IBM Corp., Armonk, NY, USA). *p* < 0.05 was set as significant. Generative artificial intelligence (AI) has been used in this article to help study the design and interpretation of statistical concepts. The information provided by AI has been checked and verified by the authors for accuracy by consulting published scientific sources.

## 3. Results

### 3.1. Global Data

Of the 340 participants, 63.20% were over 85 years old, 28.23% were male, and the median LOS was 24 months (range: 6–119 months) (Table 1). Most participants had common age-related comorbidities, such as DM, hypertension, and dyslipidaemia.

The regular Mediterranean diet accounted for 83.30% of the diets given to the participant in the 10 nursing homes. Among the 340 older adults, only 40 participants (13.70%) were receiving oral nutritional supplements. Overall, the mean EQ-VAS reflected a good self-perceived health status.

### 3.2. Nutritional Risk and Disease-Related Malnutrition Assessment

**Figure 1:**
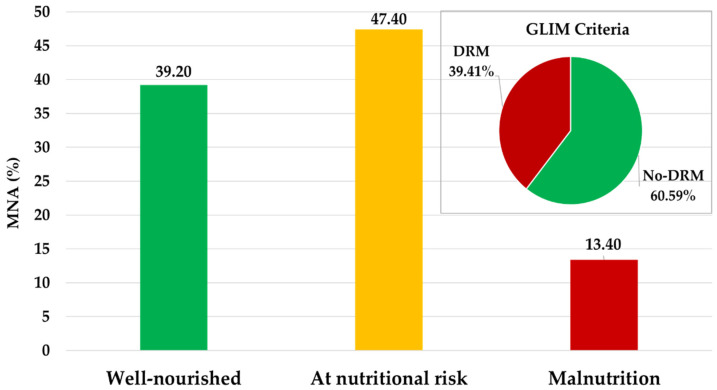
Prevalence of nutritional risk and disease-related malnutrition in 340 older nursing home residents (Results shown in bars were obtained from the Mini-Nutritional Assessment (MNA) questionnaire. The pie chart represents the results of applying the Global Leadership Initiative in Nutrition (GLIM) diagnostic criteria. DRM, disease-related malnutrition; MNA, Mini-Nutritional Assessment).

Nutritional risk and malnutrition, as screened by the MNA, were 60.8% (Figure 1). MNA was correlated positively with BCM (*r* = 0.26; *p* < 0.001), resistance (*r* = 0.25), MM (*r* = 0.23; *p* < 0.001), and EQ-VAS (*r* = 0.22; *p* < 0.001) but inversely with weight loss % (*r* = −0.27) (*both*, *p* < 0.001).

The global prevalence of DRM in the cohort was 39.41% (*n* = 134). Weight loss (%) correlated negatively with MAMC% (*r* = −0.21; *p* = 0.027), reactance (*r* = −0.17; *p* = 0.002), and BCM (*r* = −0.16; *p* = 0.010) and resistance (*r* = −0.33; *p* < 0.001). Serum CRP was directly correlated with ECW (*r* = 0.18; *p* = 0.010), and inversely with resistance (*r* = −0.25; *p* = 0.005), reactance (*r* = −0.20; *p* = 0.008), and serum albumin (*r* = −0.62; *p* < 0.001).

### 3.3. Comparison Between Groups According to GLIM Criteria

Table 2 summarises clinical, nutritional, and laboratory data of 340 participants according to GLIM diagnosis. Mean age and LOS did not differ, whereas, as expected, the DRM group had significantly lower MNA scores. Frailty risk was identified in 196 residents (57.10%), with higher prevalence in the DRM group *versus* the No-DRM group (74.62% vs. 46.60%; *p* = 0.034).

The mean BMI was significantly consistent with overweight, with non-significant mean TSF values. MAMC% and CC were both markedly lower in DRM (at least, *p* < 0.05).

Similarly, BIA-derived measurements, such as TBW and ECW, were significantly increased, whereas low resistance and PhA values were seen in DRM-diagnosed older adults compared with the No-DRM group (Table 2).

**Figure 2:**
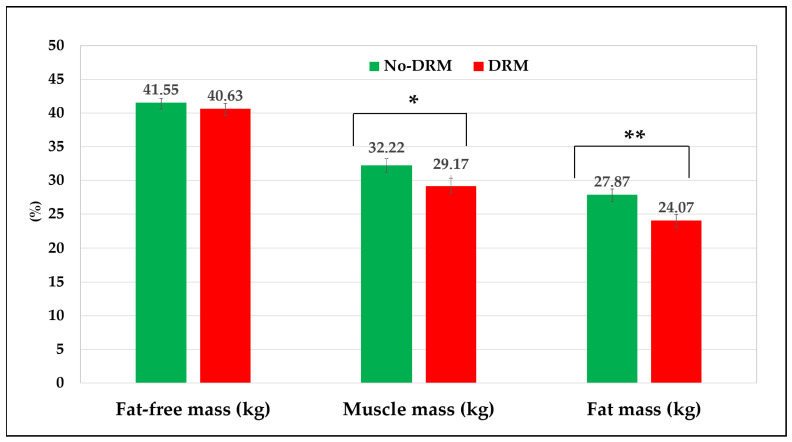
Body composition parameters in 340 nursing home residents. Values are expressed in percentages (%). *p*-values are based on the Chi-square test. * *p* = 0.007; ** *p* = 0.023.

Figure 2 shows body composition measurements assessed by BIA in the study. The DRM group had significantly lower mean values of MM and FM, whilst FFM was slightly similar to the No-DRM group (*p* = 0.340).

Table 3 displays the clinical and laboratory parameters of 340 nursing home residents. Older people with DRM had significantly lower concentrations of haemoglobin, serum albumin, and transferrin, whilst significant median differences with CRP were observed in comparison with non-DRM participants, respectively (1.04 vs. 1.68; *p* = 0.004). The DRM group’s lower scores on the EQ-VAS showed poorer self-perceived health status than the non-DRM group, reflecting substantial differences in self-rated health (Table 3).

### 3.4. Binary Logistic Regression Analysis

Results from multivariate regression analysis are shown in Table 4. A cut-off of ≥3 points on the FRAIL questionnaire was significantly associated with an increased likelihood of malnutrition (OR: 3.317; 95%CI: 1.456–7.556), whereas BIA-derived measurements such as MM (OR: 0.732), PhA (OR: 0.033), serum albumin (OR: 0.070) and better perceived health status (EQ-VAS) were identified as significant protective factors against DRM (*at least*, *p* < 0.05).

## 4. Discussion

The results of the study demonstrate a high prevalence of nutritional disorders, highlighting the high vulnerability to developing malnutrition in the older population living in nursing homes. Older adults with DRM had higher CRP levels, lower MM, and greater frailty, suggesting an interaction between low-grade systemic inflammation, deterioration in body composition, and loss of MM. These findings are consistent with previous evidence [4,9,22,23] linking DRM to poorer health and an increased risk of adverse clinical events. In this regard, early identification of DRM using the GLIM criteria is useful for preventing the progression of nutritional and functional deterioration in institutionalised older adults.

Nutritional disorders are multifactorial health concerns in older people. Poor nutritional status has been associated with reduced food intake, continuous weight loss, inflammation, frailty, sarcopenia, and cognitive impairment, leading to functional decline and increased morbidity [10,14,32]. Specifically, in this Mediterranean cohort, a high frequency of very old people, common age-related comorbidities, and a moderately good self-perceived health status were observed, with no differences in terms of LOS (Table 2). Notably, this cohort of institutionalised older adults did not have cognitive impairment and was functionally independent. Therefore, this study partially assessed the hidden nutritional burden of residents who were supposedly in better health.

The MNA questionnaire is a validated tool strongly associated with morbidity and mortality in older adults [3,8,12,33]. In this cohort, nutritional risk evaluated by the MNA was 47.4% and malnutrition accounted for 13.4%, highlighting its utility for early intervention in older adults. Findings from the current study align with large European studies [5,10,13,16,22,34], which report nutritional risk ranging from 30% to 60%.

According to the selected GLIM criteria, 38.90% were diagnosed with DRM. So far, only a few studies have applied GLIM criteria among nursing home residents. A longitudinal study also conducted in a Mediterranean cohort [9] examined the association between various GLIM models and mortality among nursing home residents. The combined adjusted 1-year follow-up survival model, including unintentional weight loss and inflammation, demonstrated that malnutrition was associated with a 2.37-fold increase in mortality rate. Further studies using different GLIM assessment criteria reported malnutrition prevalence rates ranging from 28.0% to 52.0% [4,13,22,23], findings in a similar way to those of our study.

Phenotypic criteria, such as weight loss over time and inflammaging, are involved in modulating appetite, energy expenditure, protein turnover, immune function, and physical performance [2,13,35,36]. In the current study, higher unintentional weight loss and reduced MAMC and CC were observed in malnourished individuals. CC was not used as a method to fulfill the phenotypic criterion of low muscle mass for GLIM diagnosis [37]. Likewise, serum CRP (since available in all participants) was also used to fulfill the etiologic disease burden/inflammation GLIM criterion for the diagnosis of malnutrition [20]. Inflammaging poses a significant risk for morbidity and mortality in older adults. Systemic inflammation increases with age and comorbidities and is linked to decline in muscle strength, muscle loss, and nutritional compromise, contributing to age-related diseases such as sarcopenia, neurodegenerative disorders, and CVD [17].

Inflammation was assessed in this study using CRP concentration ≥ 1 mg/dL as an indicator of mild or subclinical systemic inflammation. Participants with acute or chronic active inflammatory diseases with clinical inflammation associated with infections, tissue damage, or autoimmune diseases related to a marked elevation of inflammatory mediators were excluded. High CRP concentrations in the current study were interpreted as a marker of low-grade metabolic inflammation, characterised by a mild and persistent immune response associated with metabolic disturbances or physiological, rather than active disease. This finding reinforces the relationship between inflammation and DRM according to GLIM criteria. Growing concern about the high prevalence and adverse health effects of both malnutrition and inflammation has prompted greater attention to the implementation of earlier nutritional interventions [17,38].

Moreover, the global prevalence of frailty risk was 57.64%. Frailty is a complex syndrome characterized by reduced physiological reserve and increased vulnerability to adverse outcomes, as was partially associated with the risk of frailty (79.20%) in the DRM group. Particularly, frailty risk in this study increased the risk of DRM by 3.317-fold (95% CI: 1.456 to 7.556; *p* = 0.004) (Table 4). Pooled results from a meta-analysis [39] demonstrated that frailty significantly increased the risk of mortality 1.88-fold (95% CI: 1.57 to 2.25; *p* < 0.001) in nursing home residents. These findings are notably higher than previous studies [39,40], showing that it is mandatory to screen regularly to prevent adverse outcomes and mortality among nursing home residents.

Additionally, the BIA-derived measurements showed high mean values of resistance, TBW, ECW, and low means of MM, PhA, and serum albumin concentrations in the DRM group, which overall converge to DRM (Table 2). Overhydration, muscle wasting, and hypoalbuminaemia are nutritional disorders that often accompany malnutrition in older adults [41].

Low PhA values have been recognized as an independent and significant nutritional indicator of worse prognosis, increasing decline in physical function, disability, sarcopenia, and mortality [42,43]. However, it should be noted that MM, PhA, and serum albumin were significant protective markers against malnutrition in the regression model (Table 4). These findings highlight the need to use a combination of nutritional markers and techniques to carry out a comprehensive nutritional assessment in older people.

Self-reported perceived health status is an indicator of overall well-being and functional capacity among nursing home residents. Several studies [4,34,44] have shown that self-perceived health status has been associated with frailty, nutritional status, and the risk of adverse clinical outcomes. Notably, the mean EQ-VAS values differed significantly according to the nutritional status in this study. Older adults with DRM had poorer self-rated health status than those without DRM. Thus, in this cohort, each unit increase in EQ-VAS was significantly associated with a 3.90% reduction in the likelihood of DRM (OR = 0.961; 95%CI: 0.926 to 0.996; *p* = 0.029). Our results are consistent with previous studies [45,46,47], suggesting that older adults with better self-reported health status have both improved nutritional status and greater functional independence.

This study has some strengths and limitations. Firstly, the strengths of this study are based on the fact that, to the best of our knowledge, this is one of the few studies published on older nursing home residents without cognitive or functional impairment using multiple nutritional assessment criteria together. Our results showed the hidden and often underdiagnosed underside of malnutrition in nursing homes included in the study. In fact, it should be noted that most of the studies available were carried out by heterogeneous cohorts (mixed community-dwelling or hospitalized samples with age-related cognitive decline and functional limitations). Secondly, this is a multicentre cross-sectional study carried out in a Mediterranean cohort from 10 Spanish nursing homes, which precludes the establishment of causal relationships between identified predictors and DRM. In this study, weight loss (phenotypic criterion) and CRP concentration (an etiological criterion) were used as GLIM diagnostic criteria. Thirdly, to date, there are scarce studies available that encompass nutritional screening and diagnosis of DRM together with body composition analysis by BIA and self-perceived health status. The absence of limited global criteria used hinders the comparability of results across studies and settings. Fourthly, as limitations of the study, it should be noted that there may have been bias in the selection and collection of data. Nonetheless, the residual confounding from unmeasured variables (e.g., socioeconomic status, medical use, sarcopenia), as well as collinearity and statistical adjustments, were tested. Fifthly, one limitation of the present study is the absence of a standardised comorbidity index, such as the Charlson index or the cumulative illness rating scale. This may limit the ability to fully adjust for the influence of comorbidity burden on the relationship between DRM, inflammation, frailty, and any other nutritional disorders. However, given that major metabolic comorbidities were recorded and participants with acute or chronic inflammatory diseases were excluded, it is considered that the potential confounding effect has been partially controlled. Sixthly, in the current study, dietary food and fluid intake were not individually recorded. The residents of the nursing home followed a Mediterranean-pattern diet, based primarily on the consumption of cereals, vegetables, fruit, legumes, olive oil, dairy products, and moderate protein intake. Lastly, the generalisation of the study results is limited to institutionalised older adults without cognitive impairment who are clinically stable and functionally independent.

## 5. Conclusions

The study identified a high prevalence of nutritional disorders among nursing home residents in the Mediterranean cohort. Older adults with DRM had greater unintentional weight loss, inflammation, and a high risk of frailty, as well as reduced muscle mass compared to those without DRM, underscoring the need for routine nutritional assessment and early nutritional intervention. Subclinical low-grade systemic inflammation is a risk factor for DRM and functional decline in older adults living in nursing homes. Further studies, along with preventive nutritional policies, are needed to ameliorate the impact of nutritional disorders on health outcomes in nursing homes.

## Figures and Tables

**Table 1:** Demographic and clinical characteristics of 340 older nursing residents.

Variables	Total (*n* = 340)
Gender (Male) *n* (%)	96 (28.23)
Age (years)	87.14 ± 6.99
LOS (months)	36.57 ± 38.70
DM *n* (%)	78.0 (25.90)
Hypertension (mm Hg) *n* (%)	260.0 (76.70)
Dyslipidemia *n* (%)	183.0 (53.82)
Standard Mediterranean diet *n* (%)	249.0 (83.30)
Oral nutritional supplements *n* (%)	40.0 (13.70)
EQ-VAS (points)	72.47 ± 18.40

DM, diabetes mellitus; EQ-VAS, EuroQol Visual Analogue Scale; LOS, length of stay.

**Table 2:** Socio-demographic, clinical, anthropometric, and body composition measures in 340 nursing home residents according to nutritional status *.

Variables	No-DRM (*n* = 206)	DRM (*n* = 134)	*p*-Value
Age (years)	87.23 ± 6.68	86.95 ± 7.51	0.717
LOS (months)	31.0 ± 28.35	30.52 ± 25.63	0.877
MNA (points)	7.74 ± 4.99	10.95 ± 4.33	<0.001
FRAIL *n* (%) ^&^	96.0 (46.60)	100.0 (74.62)	0.034
Body weight (kg)	67.42 ± 12.83	62.27 ± 13.47	0.001
BMI (kg/m^2^)	28.81 ± 4.90	26.28 ± 5.41	<0.001
Weight loss (%)	1.63 ± 8.58	7.37 ± 8.04	<0.001
TSF (mm)	21.01 ± 8.58	18.71 ± 8.17	0.100
MAMC (%) ^‡^	94.86 ± 17.06	90.51 ± 14.70	0.027
CC (cm)	33.47 ± 4.28	31.80 ± 4.13	0.001
Resistance (R)	559.65 ± 91.22	533.64 ± 80.25	0.015
Reactance (ꭕc)	62.57 ± 13.44	55.46 ± 15.19	0.257
Exchangeable Na/K	0.89 ± 0.18	0.92 ± 0.23	0.070
TBW (L)	33.12 ± 8.10	36.02 ± 9.80	0.015
ECW (L)	14.23 ± 3.01	15.32 ± 4.75	0.045
ICW (L)	19.30 ± 4.72	18.35 ± 4.11	0.103
BCM (kg)	23.58 ± 4.86	22.94 ± 5.23	0.322
PhA (°)	6.46 ± 1.13	5.23 ± 1.17	0.001

*p*-Values are based on the Chi-square or Student’s *t*-test. * Nutritional status was classified according to the GLIM criteria, using the phenotypic criterion of weight loss ≥ 5% in the last 6 months in combination with the etiological criterion of serum CRP ≥ 1 mg/dL. ^&^ Frailty risk was measured by the FRAIL questionnaire [31]. ^‡^ Mid-arm muscle mass circumference (MAMC) was compared with anthropometric reference values at the 50th percentile for the older Spanish population [28]. BMI, body mass index; BCM, body cell mass; CC, calf-circumference; DRM, disease-related malnutrition; ECW, extracellular water; ICW, intracellular water; LOS, length of stay; MAMC, mid-arm muscle circumference; MNA, Mini-Nutritional Assessment; PhA, phase angle; TBW, total body water; TSF, triceps skinfold thickness.

**Table 3:** Laboratory parameters of 340 participants in the study *.

Variables	No-DRM (*n* = 206)	DRM (*n* = 134)	*p*-Value
Haemoglobin (g/dL)	12.56 ± 1.42	11.71 ± 1.60	<0.001
Total lymphocyte count (×10^3^/mm^3^)	1977.05 ± 623.93	1794.75 ± 677.58	0.060
Albumin (g/dL)	3.90 ± 0.35	3.74 ± 0.32	<0.001
Transferrin (mg/dL)	219.96 ± 29.04	189.37 ± 33.01	<0.001
CRP (mg/dL)	1.04 (2.41)	1.68 (1.98)	0.004
EQ-VAS (points)	73.26 ± 18.57	67.34 ± 16.57	0.036

CRP values are expressed as a median and interquartile range (IQR). * *p*-Values are based on Chi-square, Student’s-test, and the Mann–Whitney U test (serum CRP). CRP, serum C-reactive protein; EQ-VAS, EuroQol Visual Analogue Scale.

**Table 4:** Multivariate logistic regression analysis in 340 nursing home residents *.

Variables	OR (95% CI)	*p*-Value
Frailty risk (≥3 points)	3.317 (1.456 to 7.556)	0.004
Muscle Mass (kg)	0.732 (0.568 to 0.944)	0.016
Phase angle (°)	0.033 (0.002 to 0.500)	0.014
Albumin (g/dL)	0.070 (0.012 to 0.394)	0.003
EQ-VAS (points)	0.961 (0.926 to 0.996)	0.029

Frailty risk was measured by using the FRAIL questionnaire [31]. * *p*-Values are based on multivariate logistic regression analysis based on GLIM criteria classification (No-DRM and DRM) as a *dummy* variable. OR, odds ratio; 95%CI, 95% confidence interval. EQ-VAS, EuroQol Visual Analogue Scale.

## Data Availability

The original contributions presented in this study are included in the article. Further inquiries can be directed to the corresponding author.

## Abbreviations

BIA	Bioelectrical impedance analysis
BCM	Body cell mass
BMI	Body mass index
BW	Body weight
CC	Calf circumference
CRP	Serum C-reactive protein
CVD	Cardiovascular disease
DM	Diabetes mellitus
DRM	Disease-related malnutrition
ECW	Extracellular water
EQ-VAS	EuroQol Visual Analogue Scale
FFM	Fat-free mass
FM	Fat mass
FRAIL	Fatigue, Resistance, Ambulation, Illnesses, and weight loss
GLIM	Global Leadership Initiative on Malnutrition
ICW	Intracellular water
LOS	Length of stay
MAC	Mid-arm circumference
MAMC	Mid-arm muscle circumference
MM	Muscle mass
MNA	Mini-Nutritional Assessment
PhA	Phase Angle
TBW	Total body water
TSF	Triceps skinfold thickness
UBW	Usual body weight
